# Arterial hypoperfusion as a negative predictive marker for primary hepatic malignancies treated with Y-90 glass microsphere transarterial radioembolization

**DOI:** 10.3389/fonc.2024.1433480

**Published:** 2024-08-07

**Authors:** Bita Kalaghchi, Semra Ince, Justin Barnes, Kendall Kiser, Re-I Chin, Justin Mikell, Shahed Badiyan, Jose Garcia, Jacqueline Zoberi, Maria Bernadette Majella Doyle, Benjamin Tan, Seung Kim, Tyler Fraum, Hyun Kim

**Affiliations:** School of Medicine, Washington University in St. Louis, St. Louis, MO, United States

**Keywords:** hepatocellular carcinoma, Y-90, intra-arterial therapy, hypoperfusion, cholangiocarcinoma

## Abstract

**Background:**

Radioembolization with yttrium-90 (Y-90) is utilized to treat primary liver malignancies. The efficacy of this intra-arterial therapy in arterially hypoperfused tumors is not known.

**Methods:**

We reviewed data of patients with primary liver tumors treated with Y-90 prescription doses of at least 150 Gy. Baseline patient characteristics, treatment history, imaging-based tumor response assessments, and clinical outcome metrics were recorded. Tumors were classified as arterially hyperperfused versus hypoperfused on post-TARE Y-90 SPECT/CTs or pre-TARE hepatic perfusion SPECT/CTs. Perfusion status was correlated with tumor response assessments and clinical outcomes. Cox proportional hazards models were utilized to compare survival and progression-free survival. Inverse probability weighting was utilized to account for clinical factors and adjusted multivariable proportional hazards analyses to examine the relationship of quantitative perfusion and cancer outcomes.

**Results:**

Of 400 Y-90 treatments, 88 patients received a prescribed dose of at least 150 Gy and had pre- or post-treatment SPECT/CT images. 11 and 77 patients had arterially hypoperfused and hyperperfused lesions, respectively. On dedicated liver MRI or CT at 3 months after Y-90, the complete response rates were 5.6% and 16.5% in the hypoperfused and hyperperfused cohort, respectively (*P* = 0.60). When controlling for various clinical features, including tumor histology, patients with arterially hypoperfused tumors had significantly shorter progression-free survival (HR 1.87, 95% CI - 1.03 - 3.37, *P* = 0.039) and greater elsewhere liver (HR 3.36, 95% CI = 1.23 - 9.20, *P* = 0.019) and distant failure (HR 7.64 (2.71 - 21.54, *P* < 0.001). In inverse probability weighted analysis, patients with arterially hypoperfused tumors had worse overall survival (P = 0.032). In the quantitative analysis, lower levels of lesion perfusion were also associated with worse clinical outcomes, again controlling for tumor histology.

**Conclusion:**

Compared to arterially hyperperfused tumors, hypoperfused primary liver tumors treated with Y-90 may have worse clinical outcomes.

## Introduction

Primary liver cancer is the seventh most common malignancy and accounts for approximately 781,000 cancer-related deaths globally each year (1). The majority of newly diagnosed patients with primary liver cancers present with advanced hepatic involvement, precluding surgical resection or focal ablative radiation therapy (2). In this setting, intra-arterial therapies such as transarterial chemoembolization (TACE) and transarterial radioembolization (TARE) with yttrium-90 (Y-90) microspheres are frequently implemented (3). TARE with Y-90 microspheres has emerged as an effective and versatile treatment for controlling and down-staging hepatic tumors (4). Although multiple prospective randomized trials have not shown any survival benefit from Y-90 TARE (5), radioembolization increases time-to-progression with fewer toxicities compared to TACE (6).

Post-TARE Y-90 bremsstrahlung imaging via single-photon emission computerized tomography (SPECT)/CT can be utilized to quantify the delivered Y-90 dose (7, 8). Y-90 SPECT/CT can reveal heterogeneity of microsphere distribution within a treated lesion and the delivered radiation dose to each part of the lesion. This information can be helpful in predicting treatment response or determining the need for early triage to additional, alternative, or adjuvant therapies (9). Prior studies have shown that the prescribed Y-90 microsphere dose correlates with tumor response to treatment (10). However, when a tumor is arterially hypoperfused as can be seen with some primary liver cancers (e.g., cholangiocarcinoma and atypical hepatocellular carcinoma), the dose delivered to the tumor is effectively reduced. Further, for tumors with areas of absent perfusion, it is unclear if these areas are completely necrotic (i.e., no viable tumor) or if they harbor sites of microscopic disease. There are no data to indicate whether treatment of arterially hypoperfused tumors with Y-90 microspheres results in similar rates of local control as can be achieved for arterially hyperperfused tumors.

To address these uncertainties, we evaluated hepatic tumor response, local control, and survival after Y-90 TARE in patients with arterially hypoperfused and hyperperfused tumors.

## Materials and methods

### Study design

We reviewed treatment of patients with primary hepatic malignancies prescribed a Y-90 dose of 150 Gy or greater was performed. Dose prescription was determined by mean dose to anatomical volume on diagnostic CT or MR imaging. The threshold for a dose to be considered segmentectomy or ablative was set at 205 Gy as per previously published experience (11, 12). All patients were treated with Y-90 glass microspheres (TheraSphere; Boston Scientific, Marlborough, MA, USA) (13).

### Y-90 TARE treatment and imaging

Approximately 1 month prior to Y-90 TARE, all patients underwent planning sessions consisting of hepatic catheter angiography with administration of Tc-99m macroaggregated albumin (MAA) into hepatic artery branches, based on the anticipated sites of subsequent Y-90 microsphere administration. During these sessions, digital subtraction angiography, with or without cone-beam CT, was used to determine the vascular supply to the tumor and to detect potential sites of extrahepatic perfusion (14). Patients then underwent Tc-99m MAA planar imaging and Tc-99m MAA SPECT/CT to determine the lung shunt fraction and to assess the distribution of intrahepatic and extrahepatic structures perfused by the selected hepatic artery branches.

The Y-90 dosage for each patient was calculated using the medical internal radiation dose equation according to the liver target treatment volumes contoured on triphasic CT or MRI scans with the use of Eclipse (Varian, Palo Alto, CA) treatment planning system (15). The prescribed dose was administered into segmental branches of the hepatic arteries for segmental treatment and the left or right hepatic artery for hemiliver treatment. Note that the branches selected for Y-90 microsphere administration were generally selected to match the branches selected for Tc-99m MAA administration during the preceding planning session. Following Y-90 TARE (typically the same or following day), most patients underwent Y-90 bremsstrahlung SPECT/CT to verify the location of delivered Y-90 microspheres.

Patients were followed with MRI every 2–4 months for the first year, and further locoregional treatments were delivered for patients with partial response, stable disease, or progressive disease at the target lesions. Failure events were categorized by site (local, regional, distant) and time. Baseline patient characteristics including all clinical data, ECOG performance status at first and last visit, delivered dose, tumor response, and any therapy before local failure were also recorded.

### Image analysis

Tumor responses were assessed on MRI using the modified Response Evaluation Criteria in Solid Tumors (mRECIST) version 1.1 for patients at 2–4 months after Y-90 TARE (16, 17). These mRECIST classifications were based on the standard-of-care clinical radiology reports. As such, interpreting radiologists had access to information from all prior imaging examinations, though the preceding Tc-99m MAA and Y-90 SPECT/CTs were not routinely reviewed during MRI interpretation.

The perfusion status of a lesion was assessed based on Y-90 SPECT/CT images (71 patients), when available, as these images reflect the actual distribution of the delivered dose relative to the target lesion(s). In such cases, the preceding Tc-99m MAA SPECT/CT images were also reviewed to confirm concordance. When Y-90 SPECT/CT images were unavailable, Tc-99m MAA SPECT/CT images (45 patients) from the pre-Y-90 planning session were utilized instead, based on the principle that the Y-90 microspheres were subsequently administered via the same hepatic arterial branches as the Tc-99m MAA.

Any available dynamic contrast-enhanced CT or MRI examinations were reviewed prior to analysis of the SPECT/CT images to confirm the location of the target lesion(s). The target lesions were assessed by two readers (4 years and 10 years of post-training experience in nuclear medicine) both qualitatively and quantitatively.

**Figure 1:**
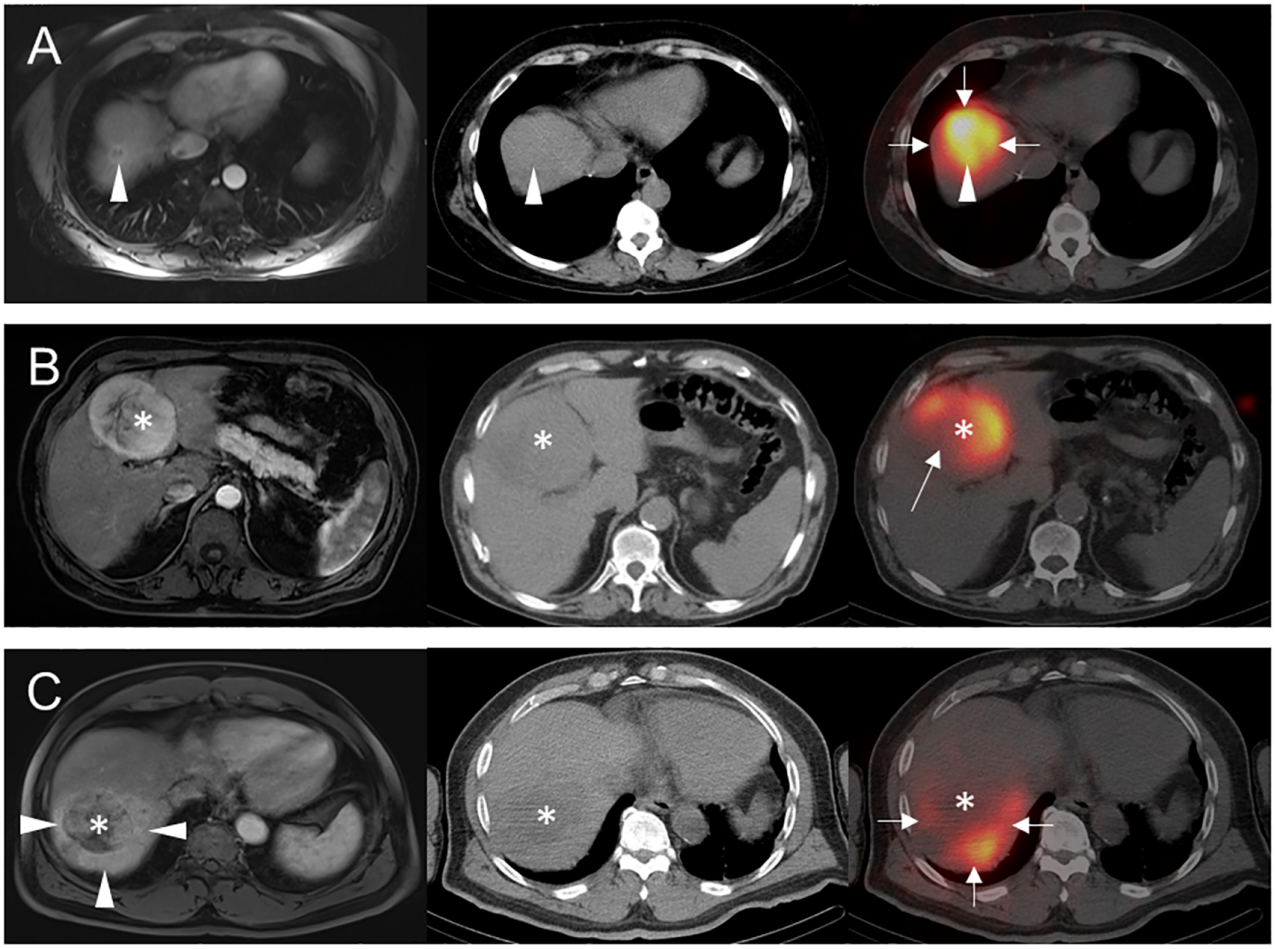
Examples of arterial perfusion categorizations. Pre-treatment axial arterial phase post-contrast T1-weighted MR images (left column) prior to Y-90 TARE and post-treatment axial noncontrast CT (middle column) and fused Y-90 SPECT/CT images (right column) are shown for 3 separate patients. **(A)** 56-year-old female with segment 8 intrahepatic cholangiocarcinoma (arrowheads) underwent Y-90 TARE, with post-treatment Y-90 SPECT/CT showing uniform activity throughout segment 8 (arrows), including the target lesion (arrowhead). This tumor was classified as hyperperfused, based on its activity similar to background liver parenchyma. **(B)** 85-year-old male with segment 4B HCC (asterisks) underwent Y-90 TARE with post-treatment Y-90 SPECT/CT showing an area of near-absent Y-90 activity (arrow) in the right posterolateral aspect of the tumor, presumably due to interval necrosis versus partial arterial supply by an unmapped hepatic arterial branch. Given these findings, the tumor was classified as hypoperfused. **(C)** 80-year-old male with segment 7 HCC (asterisks) underwent Y-90 TARE with post-treatment Y-90 SPECT/CT showing tumor activity substantially less than background (arrows), potentially related to peritumoral shunting as seen on prior MRI (arrowheads). Based on these findings, the tumor was classified as hypoperfused.

For the qualitative analysis, a case was classified as arterially hypoperfused if all or part of the target lesion(s) had visually less activity than the surrounding uninvolved liver parenchyma. A case was considered arterially hyperperfused if the target lesion(s) had activity visually *similar or greater* than the background liver. When lesions had necrotic areas on preceding CT or MRI (3 patients), lack of activity in these areas was considered arterial hypoperfusion, since these areas without macroscopic viable tumor could still contain viable microscopic malignancy. When a portion of the target lesion(s) extended outside the treatment territory and consequently contained no activity (5 patients), such lesions were considered *intrinsically* hyperperfused but *functionally* hypoperfused given the failure of the Y-90 TARE to treat a portion of macroscopic viable tumor. Note that these 5 patients were included with other arterially hyperperfused cases in the main analysis. When activity was seen only in the lesion (i.e., minimal-to-no background parenchymal activity), the lesion was considered arterially hyperperfused. Example cases illustrating these classifications are shown in **Figure 1**.

Quantitative analysis was performed for the subset of patients (101/116; 87%) with available attenuation-corrected SPECT images from either Y-90 SPECT/CT or Tc-99m SPECT/CT by two authors with expertise in nuclear medicine (XX, YY). A circular two-dimensional region of interest (ROI) was placed on a representative slice of the target lesion (or in cases of multiple lesions, the dominant target lesion). An ROI of similar size and shape was placed in the background liver on the same slice, adjacent to the target lesion. The mean value of each ROI was extracted and utilized to generate lesion-to-background ratios (LBRs), defined as the mean value of the lesion ROI divided by the mean value of the background liver ROI, on a per-patient basis. Note that arterially hyperperfused versus hypoperfused status was based on the visual analysis described above rather than on the LBRs.

### Statistical analysis

Summary statistics of the characteristics of the study population were generated. Response rates were evaluated using contingency tables, limited to patients with at least 3 months of follow up after the Y-90 TARE. We evaluated overall survival (OS) and progression-free survival (PFS) using the Kaplan-Meier method and compared OS and PFS between patients with arterially hypoperfused versus hyperperfused target lesions using the log-rank test. Local failure, elsewhere liver failure, regional failure, and distant failure were evaluated using cumulative incidence methods to account for death as a competing risk (15).

To account for potential dissimilarities in baseline patient characteristics, we conducted inverse probability (of arterial hypoperfusion) weighted (IPW) analyses based on propensity scores, which were generated via logistic regression with the following covariates: primary site (liver vs. other), histology, target volume, delivered dose, performance status, and age. Covariate balance was assessed by evaluating propensity scores (**Supplementary Figure 1**) and standardized mean differences with and without IPW adjustment (**Supplementary Figure 2**). Other classifiers were considered (e.g., LASSO regression, random forests), but logistic regression provided the best baseline covariate balancing based on standardized mean differences (**Supplementary Figure 2**). The inverse probability weights were applied to log-rank tests of OS and PFS, and further to Cox proportional hazards (OS and PFS) and Fine and Gray proportional hazards (local failure, elsewhere liver failure, regional failure, and distant failure; to account for death as a competing risk) models. Additionally, we utilized multivariable Cox and Fine & Gray proportional hazards models with the aforementioned covariates, without IPW adjustment.

Sensitivity analyses were conducted by allocating the *intrinsically* hyperperfused but *functionally* hypoperfused cases (5 patients) to the hypoperfused cohort, rather than to the hyperperfused cohort as in the main analysis. Finally, we conducted analyses evaluating the association of the survival and failure outcomes with quantitative estimates of the degree of perfusion as measured by LBRs, which were analyzed as a continuous variable. LBRs were not used to dichotomize tumors on the basis of perfusion status. All analyses were conducted using R version 4.2.1. P values are two-sided (when applicable), and an α of 0.05 was used to define statistical significance.

## Results

**Figure 2:**
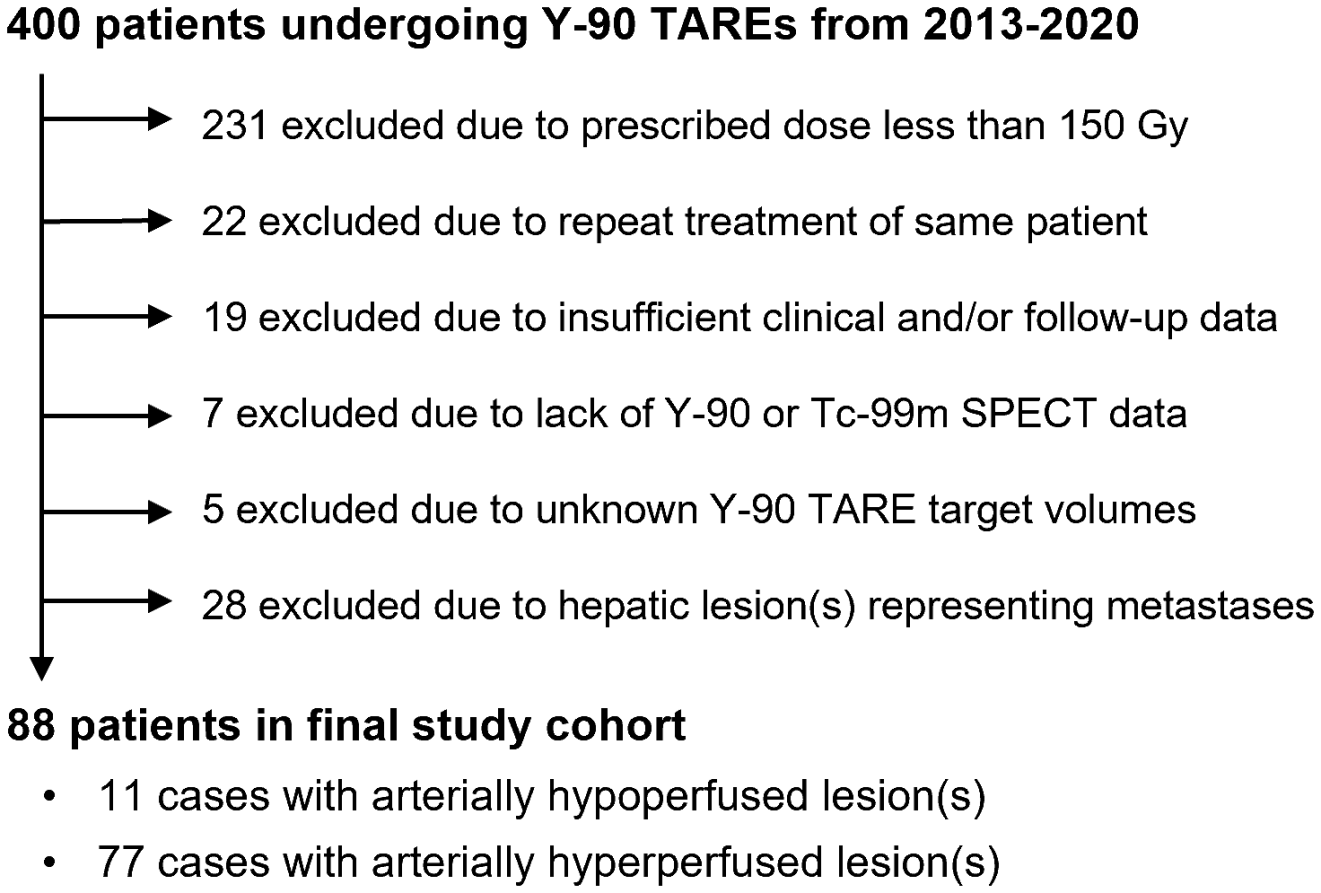
Flowchart for study cohort.

There were 400 Y-90 treatments between 2013 -2020, with 169 treatments involving prescribed doses of 150 Gy or higher. Exclusion criteria included: metastatic lesion (n=28), repeat treatments (n=22), insufficient clinical and/or follow-up data (n=19), lack of Tc-99m MAA or Y-90 SPECT/CT images for analysis (n=7), and unknown Y-90 TARE target volumes (n=5). Finally, among the final 88 patients in the cohort (**Figure 2**), there were 11 patients (12.5%, 11/88) in the arterially hypoperfused cohort and 77 patients (87.5%, 77/88) in the arterially hyperperfused cohort. Segmental dosing was prescribed in 36 of 88 patients, with 3 of the 36 ablative dosed patients demonstrating hypoperfused tumors. The mean age at treatment was 67 years, the majority of patients had a hepatocellular carcinoma (75.0%), and the mean target volume was 655 cc (**Table 1**). Mean dose prescribed per treatment was 199.1 ± 61.0 Gy for the entire patient cohort. Mean dose prescribed to hyperperfused and hypoperfused tumors were 200.5 ± 61.9 Gy and 197.8 ± 43.6 Gy, respectively.

**Table 1:** Characteristics of the study population.

		Total	Arterially hyperperfused	Arterially hypoperfused
		No. (%)		
		88 (100)	77 (100)	11 (100)
Target liver lesion histology	Hepato-cholangiocarcinoma	5 (5.7)	4 (5.2)	1 (9.1)
	Cholangiocarcinoma	17 (19.3)	14 (18.2)	3 (27.3)
	Hepatocellular carcinoma	66 (75)	59 (76.6)	7 (63.6)
Prescribed dose	<205 Gy	52 (59.1)	44 (57.1)	8 (72.7)
	≥205 Gy	36 (40.9)	33 (42.9)	3 (27.3)
ECOG	0	34 (38.6)	31 (40.3)	3 (27.3)
	1	45 (51.1)	37 (48.1)	8 (72.7)
	2	8 (9.1)	8 (10.4)	0 (0)
	3	1 (1.1)	1 (1.3)	0 (0)
Race	Asian	4 (4.5)	4 (5.2)	0 (0)
	Black	13 (14.8)	10 (13)	3 (27.3)
	White	71 (80.7)	63 (81.8)	8 (72.7)
Sex	Female	32 (36.4)	32 (41.6)	0 (0)
	Male	56 (63.6)	45 (58.4)	11 (100)
		Mean (SD)		
	Age at treatment	67.3 (11.5)	67.4 (11.9)	67.3 (8.2)
	Target volume	655.1 (442.6)	629.7 (444.9)	833.3 (400.1)

**Figure 3:**
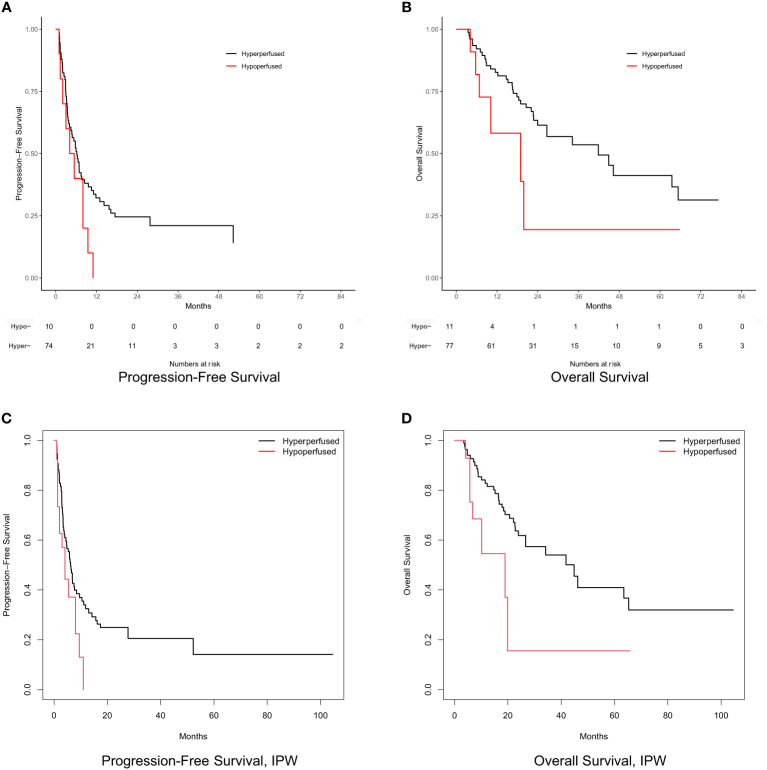
Progression-free survival and overall survival by perfusion status. **(A)** Progression-free survival. **(B)** Overall survival. **(C)** Inverse probability weight adjusted progression-free survival. **(D)** Inverse probability weight adjusted overall survival. Numbers at risk not shown for survival curves based on weighted samples **(C, D)**.

**Figure 4:**
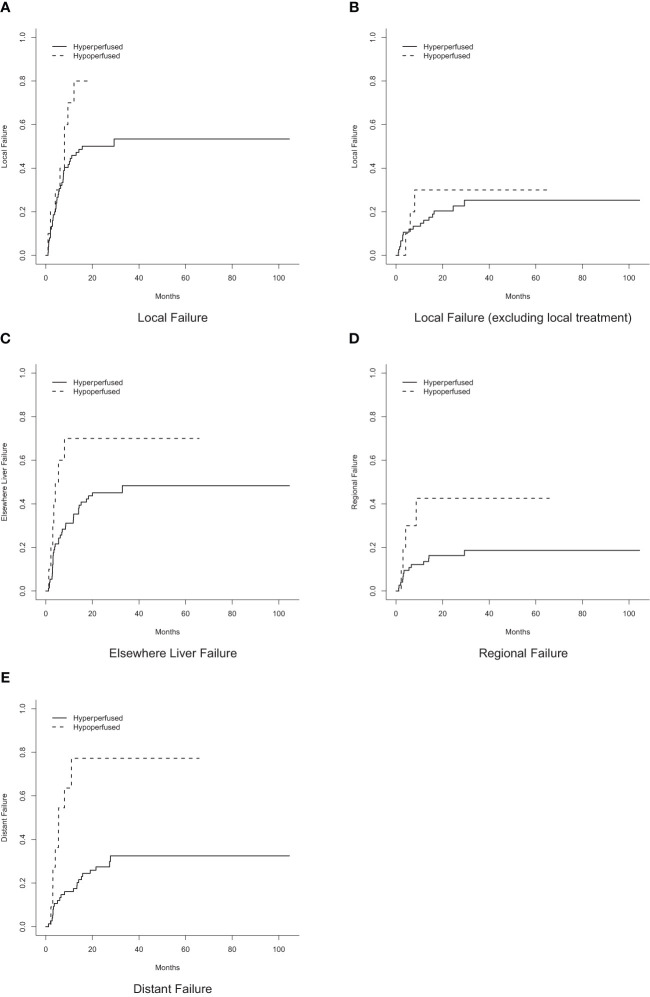
Cumulative incidence of failure by perfusion status. **(A)** Local failure. **(B)** Local failure, excluding local treatment. **(C)** Elsewhere liver failure. **(D)** Regional failure. **(E)** Distant failure.

Tumor response at 3 months on MR imaging did not differ significantly by perfusion status (*P* = 0.48) (**Table 2**). Numerically, the complete response rate was approximately one third (5.6% vs. 16.5%) in the arterially hypoperfused cohort, with similar progressive disease rates (11.1% vs 9.3%). In IPW analyses, patients with arterially hypoperfused tumors had worse overall survival (*P* = 0.032; **Figure 3**). In proportional hazards regression analyses, patients with arterially hypoperfused tumors had significantly shorter progression-free survival (HR 1.87, 95% CI - 1.03 - 3.37, *P* = 0.039), and greater elsewhere liver (HR 3.36, 95% CI = 1.23 - 9.20, *P* = 0.019) and distant failure (HR 7.64 (2.71 - 21.54, *P* < 0.001), with mixed results across analyses for local failure (**Table 3**, **Figures 3**, **4**). Results were similar in a sensitivity analysis, which included *intrinsically* hyperperfused but *functionally* hypoperfused tumors with the hypoperfused cohort (**Supplementary Tables 1, 2**). There were no differences in response based on a dose threshold of 205 Gy in the hyperperfused (p=0.71) and hypoperfused (p=0.23) cohorts.

**Table 2:** Clinical tumor response at 3 months (mRECIST 1.1).

	Arterially hyperperfused	Arterially hypoperfused
Complete response	16 (16.5)	1 (5.6)
Partial response	30 (30.9)	6 (33.3)
Stable disease	21 (21.6)	2 (11.1)
Progressive disease	9 (9.3)	2 (11.1)

Chi-square test: *P* = 0.60.

**Table 3:** Overall survival, progression-free survival, and failure rates for tumors with arterial hyperperfusion vs hypoperfusion.

	Univariable Analysis, Log-Rank Tests^a^		Proportional Hazards Models^c^			
	*P* value		Multivariable analysis		Inverse probability weighting	
	Without IPW Adjustment	With IPW Adjustment^b^	HR^c^ (95% CI)	*P* value	HR^c^ (95% CI)	*P* value
Overall survival	0.095	0.032	2.2 (0.76 - 6.35)	0.145	2.46 (0.94 - 6.47)	0.067
Progression-free survival	0.081	0.060	1.87 (1.03 - 3.37)	0.039	1.95 (1.1 - 3.43)	0.021
Local failure (without considering therapy prior to LF)	0.60	NA	1.81 (0.52 - 6.27)	0.352	1.41 (0.4 - 4.94)	0.594
Local failure (including local therapy)	0.10	NA	2.28 (1.07 - 4.89)	0.033	1.66 (0.82 - 3.37)	0.162
Elsewhere liver failure	0.040	NA	3.36 (1.23 - 9.2)	0.019	2.81 (1.06 - 7.41)	0.037
Regional failure	0.087	NA	3.08 (0.9 - 10.53)	0.072	2.34 (0.72 - 7.58)	0.155
Distant failure	<.001	NA	7.64 (2.71 - 21.54)	<.001	4.12 (1.64 - 10.35)	0.003

a *P* values for OS and PFS are based on the log-rank test based on Kaplan-Meier survival curves. The *P* values for the other statistics are based on tests of the cumulative incidence.

b Inverse probability weighted cumulative incidence function is not well-defined. See Proportional Hazards Model (Fine-Gray) results for adjusted/multivariable *P*-values.

c HR represents relative risk of event for hypoperfused relative to hyperperfused tumors. HR and associated *P* values for OS and PFS are based on the Cox proportional hazards model with robust standard errors. The other estimates are subdistribution hazard ratios from the Fine and Gray proportional hazards model for competing risks.

Quantitative estimates of perfusion (i.e., LBRs), which were analyzed as a continuous rather than dichotomized variable, were significantly associated with local failure, elsewhere liver failure, and distant failure in adjusted analyses (**Supplementary Table 3**). In one case, a tumor was classified as arterially hyperperfused (on the basis of activity similar to the background liver parenchyma) but found to have an LBR less than 1.0 (in this case, 0.77). Otherwise, all tumors classified as arterially hyperperfused had LBRs ≥1.0, and all tumors classified as arterially hypoperfused had LBRs <1.0. In other words, there was near-perfect agreement between the qualitative and quantitative analyses, though perfusional status as a dichotomized variable was based only on the qualitative (i.e., visual) analysis.

## Discussion

To our knowledge, this work is the first to evaluate the efficacy of Y-90 microspheres for hypoperfused primary liver malignancies. These data indicate that arterially hypoperfused tumors, defined either visually or quantitatively, may have decreased overall survival and increased local recurrence after intra-arterial Y-90 microsphere administration. Furthermore, patients with arterially hypoperfused tumors demonstrated numerically half the clinical complete response and nearly double the disease progression. Although there was no significant difference in mRECIST assessment between groups, this finding may have been due to the limited number of patients in this retrospective review. Overall, these data are important for clinical decision making, as some clinicians may choose Y-90 for patients with hypoperfused tumors with the assumption that poor arterial perfusion is equivalent to necrosis or acellular fibrosis with no viable tissue. However, it is possible that these areas of arterial hypoperfusion harbor microscopic disease that will not be exposed to sufficiently high concentrations of Y-90 to achieve ablative or near-ablative radiation doses. More specifically, as the beta particles emitted by Y-90 travel (on average) only 2.5 mm through tissue, radiation emitted from arterial hyperperfused portions of the tumor will presumably not result in adequately high radiation doses in the arterially hypoperfused portions (18).

Prior work demonstrated that tumor vascularity may not significantly influence outcomes in Y-90 treatment of secondary liver malignancies (metastatic disease) with hemiliver doses of 120 Gy (19). However, increasing data indicate that higher doses are needed for improved hepatic control (20). Thus, the lack of difference in treatment outcomes between hypervascular and hypovascular tumors may have been due to insufficient treatment of the malignancies rather than a true lack of impact of tumor vascularity. Future studies with ablative segmentectomy dosing are warranted in the metastatic setting to corroborate previously published findings.

Y-90 TARE is occasionally not feasible due to excessive lung shunt fraction, multiple feeder vessels to the tumor, or off-target perfusion of extrahepatic abdominal organs (13, 21, 22). Although dysmorphic intratumoral vessels may indicate higher lung shunt, predictors for high lung shunting are generally not well defined without intraarterial mapping (23, 24). Our data suggest that arterial hypoperfusion of the target lesion(s), as assessed on a Tc-99m MAA SPECT/CT at the time of Y-90 planning, might constitute a relative contraindication for Y-90 TARE. External beam radiation therapy can deliver radiation to the entire tumor irrespective of its perfusion, and theoretically may be associated with improved control of hypoperfused tumors. Therefore, SBRT may be considered as an alternative treatment strategy in this setting. Although this recommendation is based on retrospective analysis, caution should be exercised before proceeding with Y-90 TARE in the setting of an arterially hypoperfused target lesion until there are additional retrospective or prospective studies indicating that Y-90 TARE is effective for hypoperfused tumors.

Our study had several limitations, including its retrospective, single-center design with a relatively small number of patients with hypoperfused tumors. It would be difficult to complete a prospective study with this clinical question given the relative infrequency of hypoperfused tumors. As such, this retrospective analysis may be the best form of evidence to guide clinical decision-making in this setting. These findings were also derived from patients who were prescribed a dose of more than 150 Gy. However, this cutoff was established as a standard dose for hemiliver treatment and therefore could be considered insufficient for local control, potentially limiting power for detecting differences between treated groups. We included different tumor histologies which may impact treatment response. Our small histologic sample sizes precluded a meaningful subgroup analysis, but differences in outcomes between cohorts persisted even when including tumor histology as a covariate. Finally, the available imaging data (i.e., Tc-99m MAA SPECT/CT and/or Y-90 SPECT/CT) were heterogeneous. Although concordance in radioactivity distribution was confirmed for patients that underwent both studies, it is possible that the actual radiation dose delivered via Y-90 TARE may not have been accurately captured when only Tc-99m MAA SPECT/CT were available for assessment. Given the retrospective nature of this study, post-treatment SPECT/CT was not always possible due to insurance coverage and patient scheduling preference (many travel far away to receive treatment at our tertiary cancer center). Since the Y-90 dose is delivered through the same hepatic arterial branch or branches as the Tc-99m MAA dose, and the concordance of distribution in patients that received both studies, we believe that using the Tc-99m MAA SPECT/CT as a surrogate for the distribution of Y-90 microsphere delivery is clinically reasonable given the limitations of this retrospective patient experience.

Important to note is that this study only evaluated treatment with glass microspheres. This is likely not a significant limitation as glass microspheres Y-90 is FDA approved for and most routinely used for hepatocellular carcinoma. However, it may be important to evaluate if similar limitations with hypoperfused tumors applies when treating with resin microspheres, where there are significantly more microspheres per treatment and prescribed activity. While it is possible that more spheres may result in more distribution, including poorly perfused regions, it is also possible that poor perfusion may limit sphere localization even when more spheres are available. Further, lower activity per sphere may limit the amount of treatment response if poor perfusion limits the number of resin microspheres, even though numerically more than glass microspheres.

Despite these limitations, these data suggest that alternate treatments, such as photon or proton external beam radiation, should be considered for arterially hypoperfused tumors on Tc-99m MAA SPECT/CT. Similarly, there is a retrospective study and clinical trial using Y-90 PET/CT dosimetry to select and guide additional treatment with SBRT to underdosed (possibly due to perfusion) regions (NCT04518748) (25,26). Future studies may include pathologic examination of arterially hypoperfused regions after Y-90 TARE to evaluate for viable disease that may result in poorer local control and other oncologic outcomes.

## Data availability statement

The datasets presented in this article are not readily available because they are confidential patient data and protected by HIPAA and institutional data use agreements. Requests to access the datasets should be directed to Hyun Kim at kim.hyun@wustl.edu.

## Ethics statement

The studies involving humans were approved by Human Research Protection Office at Washington University in St. Louis School of Medicine. The studies were conducted in accordance with the local legislation and institutional requirements. The ethics committee/institutional review board waived the requirement of written informed consent for participation from the participants or the participants’ legal guardians/next of kin because it was a retrospective review of medical records.

## Author contributions

BK: Conceptualization, Data curation, Formal analysis, Investigation, Methodology, Writing – original draft, Writing – review & editing. SI: Conceptualization, Data curation, Formal analysis, Investigation, Methodology, Writing – review & editing. JB: Formal analysis, Methodology, Software, Writing – review & editing. KK: Project administration, Writing – review & editing. R-iC: Investigation, Writing – review & editing. JM: Data curation, Investigation, Methodology, Writing – review & editing. SB: Data curation, Investigation, Methodology, Writing – review & editing. JG: Data curation, Investigation, Methodology, Writing – review & editing. JZ: Data curation, Investigation, Methodology, Writing – review & editing. MD: Investigation, Writing – review & editing. BT: Investigation, Writing – review & editing. SK: Investigation, Writing – review & editing. TF: Conceptualization, Data curation, Formal analysis, Investigation, Methodology, Project administration, Resources, Supervision, Writing – original draft, Writing – review & editing. HK: Conceptualization, Data curation, Formal analysis, Investigation, Methodology, Project administration, Resources, Supervision, Writing – original draft, Writing – review & editing.
