# Supplementary material for: Arterial hypoperfusion as a negative predictive marker for primary hepatic malignancies treated with Y-90 glass microsphere transarterial radioembolization
Source: Front Oncol. 2024 Aug 7;14:1433480. doi: 10.3389/fonc.2024.1433480 (PMC11335672; doi:10.3389/fonc.2024.1433480)
Supplement: Supplementary file 1 [file DataSheet_1.docx]

Supplementary Results

Supplementary Table 1: Sensitivity Analysis of Clinical Tumor Response at 3 Months (RECIST 1.1) Including Functionally Hypoperfused Tumors

|  | Hyper-/iso-perfusion | Hypoperfusion |
| --- | --- | --- |
| Complete Response | 16 (16.5) | 1 (5.6) |
| Partial Response | 28 (28.9) | 8 (44.4) |
| Stable Disease | 21 (21.6) | 2 (11.1) |
| Progressive Disease | 8 (8.2) | 3 (16.7) |

Chi-square test P=.23

Supplementary Table 2: Sensitivity Analysis of Oncologic Outcomes Including Functionally Hypoperfused Tumors

|  | Univariable Analyses | | Multivariable^a^ | | IPTW | |
| --- | --- | --- | --- | --- | --- | --- |
|  | Unadjusted | IPW-adjusted | HR^b^ (95% CI) | P-value | HR^b^ (95% CI) | P-value |
| OS | 0.14 | 0.041 | 1.86 (0.73 - 4.69) | 0.192 | 2.27 (1 - 5.17) | 0.051 |
| PFS | 0.41 | 0.14 | 1.56 (0.84 - 2.9) | 0.157 | 1.62 (0.9 - 2.94) | 0.11 |
| Local Failure (without considering therapy prior to LF) | 0.88 | NA | 1.4 (0.41 - 4.86) | 0.593 | 1.23 (0.34 - 4.49) | 0.751 |
| Local Failure (including local therapy) | 0.43 | NA | 1.8 (0.78 - 4.11) | 0.166 | 1.47 (0.69 - 3.16) | 0.318 |
| Elsewhere Liver Failure | 0.24 | NA | 2.45 (0.89 - 6.73) | 0.081 | 2.37 (1 - 5.65) | 0.051 |
| Regional Failure | 0.03 | NA | 3.47 (1.01 - 11.94) | 0.048 | 3.31 (1.14 - 9.6) | 0.028 |
| Distant Failure | <.001 | NA | 7.57 (2.64 - 21.69) | <.001 | 4.17 (1.78 - 9.78) | 0.001 |

^a^Models are adjusted for age, performance status, diagnosis (HCC, cholangiocarcinoma, biphenotypic), target volume, and delivered dose.

^b^HR and associated P-values for OS and PFS are based on the Cox proportional hazards model with robust standard errors. The other estimates are subdistribution hazard ratios from the Fine and Gray proportional hazards model for competing risks.

Supplementary Table 3: Sensitivity Analyses of Association of Quantitative Estimates of the Degree of Perfusion as Measured by Lesion to Background Ratios with Oncologic Outcomes

|  | Hazard Ratio^a,b,c^ (95% CI) | P-value |
| --- | --- | --- |
| OS | 0.78 (0.46 - 1.32) | 0.355 |
| PFS | 0.9 (0.69 - 1.17) | 0.427 |
| Local Failure (excluding local therapy) | 0.6 (0.37 - 0.99) | 0.045 |
| Local Failure (including local therapy) | 0.8 (0.55 - 1.15) | 0.227 |
| Elsewhere Liver Failure | 0.47 (0.29 - 0.76) | 0.002 |
| Regional Failure | 0.82 (0.49 - 1.38) | 0.461 |
| Distant Failure | 0.37 (0.16 - 0.82) | 0.014 |

^a^Models are adjusted for age, performance status, diagnosis (HCC, cholangiocarcinoma, biphenotypic), target volume, and delivered dose

^b^Hazard ratios represent the change in hazard ratio for a 1 unit increase in the natural logarithm of the Lesion:Background ratio.

^c^HR and associated P-values for OS and PFS are based on the Cox proportional hazards model with robust standard errors. The other estimates are subdistribution hazard ratios from the Fine and Gray proportional hazards model for competing risks.

Supplementary Figure 1: Kernel density plots of propensity scores with and without inverse probability weighting.

Supplementary Figure 2: Standardized mean differences with and without inverse probability weighting (IPW).

Legend: Note that all patients in the hypoperfused cohort had ECOG 0-1 as compared to 88% in the hyperperfused cohort, so weighting and standardized mean difference for this factor was limited.
